# Effect of Mindfulness on Empathy and Self-Compassion: An Adapted MBCT Program on Filipino College Students

**DOI:** 10.3390/bs10030061

**Published:** 2020-02-27

**Authors:** Reginald Paul R. Centeno, Karina Therese G. Fernandez

**Affiliations:** Ateneo Bulatao Center, Ateneo de Manila University, Quezon City 917, Philippines; kfernandez@ateneo.edu

**Keywords:** mindfulness, MBCT, adapted MBCT, empathy, self-compassion, Filipinos, adolescents, college students

## Abstract

Attending college is meaningful for many young adults. This period is marked by physical, emotional, and psychological changes that can have both positive and negative effects on college students. The last two decades have seen an alarming increase in the number of college students who suffer from mental health conditions, such as depression, suicide, anxiety, and alcohol abuse. It is recommended that actions to support the students’ wellbeing must be creative and evidence-based. Research suggests that a mindfulness-based intervention may be an effective strategy to address mental health conditions among college students. This study was done to examine the efficacy of an adapted mindfulness-based cognitive therapy (MBCT) program that was implemented in a classroom setting in the Philippines and to explore how mindfulness practice can affect empathy and self-compassion on senior Filipino college students aged 19–22 years old. Two classes were used to compare the effects of mindfulness intervention. One class underwent the adapted MBCT program while the other class underwent the same kind of class without mindfulness interventions. Self-report measures of the Five Facet Mindfulness Questionnaire, Perspective Taking subscale and Empathic Concern subscale of Interpersonal Reactivity Index, and Self-compassion scale—short form were administered before undergoing the adapted MBCT and after the program. After going through the adapted MBCT, college students’ mindfulness significantly improved. Empathy and self-compassion also significantly improved after undergoing the program. This corroborates previous studies done on mindfulness and its efficacy with adolescents and suggests how practicing mindfulness can improve empathy and self-compassion with Filipino college students. It provides a promising groundwork for the emerging interest and research in Asia, particularly in the Philippines, on how the practice of mindfulness can help with the mental health of college students.

## 1. Introduction

Research on mindfulness is constantly expanding, and findings suggest numerous positive outcomes. The practice of mindfulness has grown and developed over the past two decades, and has its roots in Buddhism [1,2,3]. From the East, mindfulness has traveled to the West, where Jon Kabat Zinn applied its philosophies to behavioral interventions for medical problems. From there, mindfulness programs have expanded and have become an intervention of choice for stress, anxiety, depression, optimal performance, and enhanced well-being.

While there have been many ways of describing mindfulness, Kabat-Zinn [4] defines mindfulness as the process of bringing a certain quality of attention to moment-by-moment experience [5]. It is a form of mental training to reduce cognitive vulnerability to reactive modes of the mind that may bring about stress, emotional problems, and interpersonal difficulties [6,7]. Mindfulness begins with bringing a certain kind of awareness to the present moment, where the individual can observe the different thoughts, feelings, and sensations as they arise. In bringing attention to one’s inner experience, an opportunity for the individual to respond with a more conscious deliberation arises. 

In the Philippines, empirical support on mindfulness is still lacking. Although the popularity of mindfulness practice has gained interest and is well-received, studies on its impact on the lives of Filipinos remain scarce. In particular, there is a need to investigate how mindfulness interventions would help Filipino adolescents, given the arising challenges and concerns observed with the youth in college. 

Entering college can be considered as an opportunity and a challenge [8]. It is a time when students transition and adjust as they go through another phase in their lives. College students experience many changes, particularly psychological, cognitive, and social through the school years [9]. There can be many stressors and even the transitioning itself is stressful. Adjustment for students in college can vary from and this can depend on their developmental stages. Some students can adjust well, while there are also students who have difficulty in coping. These students can experience various problems in their academics as well as psychological issues. Sometimes, difficulty in adjusting to these changes can lead to depression and anxiety [10]. In fact, there are statistics suggesting a rise in psychological concerns among college populations [11,12]. With the different challenges and increases in reported psychological concerns with college students, the need for interventions for individuals as well as group-based settings has increased [13,14]. 

A. Mindfulness-Based Cognitive Therapy and Adolescents

Over the years, an increase in psychological concerns among college students has been observed [15]. There has been a search for empirically-based interventions that can address this need. As a result, mindfulness-based interventions have been introduced and were found to be effective [13,16,17]. Given how mindfulness has been found to have positive outcomes with adults, mindfulness interventions have also been found to help decrease stress in college students [18] as well as lessen problematic behaviors, such as their alcohol problems [19].

One particular form of mindfulness intervention that has been getting a lot of interest is mindfulness-based cognitive therapy (MBCT). MBCT [20] is an intervention originally designed to prevent relapse in depression. MBCT makes use of various techniques, including the body scan, mindful stretching, and mindful breathing, among others. It also involves the practice of becoming aware of one’s own thoughts, feelings, and body sensations as they arise, then shifting attention to the present moment [21]. This training of becoming aware of the inner experience without responding automatically allows individuals to gain skills to respond better [20]. A related practice is learning to use words or phrases during meditation to label emotional states (e.g., “here is anger”). MBCT allows people to become more aware of their thoughts and feelings and to bring them to a wider perspective [22]. Mindfulness training allows people to decenter themselves from certain inner experiences without avoiding them or suppressing them [23] (p. 34). 

MBCT has been shown to be effective for adolescents and young adults. Kaviani, Javaheri, and Hatami [24] discovered how participating in MBCT reduced depression and anxiety in college students. Thomas [25] studied how students in Malaysia who participated in MBCT were found to have decreased perceived stress. Frewen, Evans, Dozois, and Partridge [26] also found that students who participated in an adapted MBCT mindfulness intervention had a decrease in rumination, and improvement in terms of being able to let go of the automatic thinking that often results in anxiety and depression. While a good number of different studies involving MBCT in a college sample were found to have significant results, studies on the efficacy of MBCT on a young Filipino population are currently being researched. 

B. Adapted Mindfulness-Based Interventions

Known mindfulness interventions, such as MBCT, have strong empirical support regarding their efficacy across different studies. Adaptations of this intervention and other mindfulness-based interventions are also increasing in popularity and interest in different areas of study. Adapting such programs offer opportunities to address more specific needs and to apply it to a more specific group of people. With the increasing empirical support on the efficacy of mindfulness-based interventions, the process of adapting them for different populations, such as adolescents, has also started to receive interest [27]. Several studies [28,29] adapted mindfulness-based interventions and explored their effects on adolescents in a school-based setting. Biegel and Brown [28] integrated mindfulness in a program done in 5 weeks for elementary school students. After being guided into various mindfulness activities for several sessions for 5 weeks, there was a reported significant effect on the students’ social skills. Bluth, Roberson, and Gaylord [29] used an adapted mindfulness intervention based on the Mindfulness-based Stress Reduction (MBSR) for 6 weeks and reported positive changes in emotional health, self-compassion, perceived stress, and life satisfaction. Another mindfulness intervention adapted for adolescents in school-based settings is Mindfulness-Based Stress Reduction for Teens [30]. Their study introduced an adapted version of MBSR to adolescents that had significant results where some program activities were reduced in length, and parts of the program were omitted. Such interventions have been observed to help with well-being, academics, attention, social relatedness, and behavioral difficulties. The efficacy studies on mindfulness interventions have also been found to have a positive effect on self-compassion and empathy.

C. Self-Compassion and Mindfulness

Cunha et al. [31] suggested that enhancing self-compassion can be beneficial to adolescents in that they will be able to face their stresses and challenges in a healthier and more balanced state. Mindfulness is an approach that is currently being explored to improve self-compassion. 

Self-compassion can be defined as the ability to hold one’s feelings of suffering with a sense of warmth, connection, concern, and self-kindness [32,33]. Neff [32] describes how self-compassion helps promote resilience against depression and anxiety while promoting positive well-being. Neff and Vonk [34] also describe how self-compassion provides greater emotional resilience and stability because self-compassion prevents critical or harsh self-evaluation. Self-compassion allows a person to be kind to themselves, allowing them better ways of facing difficult situations and failures. Being self-compassionate helps the individual to face thoughts and emotions with themselves in a healthier manner [35], not trying to strive for impossible standards, nor self-criticizing if one falls short. 

In mindfulness training, individuals are invited to open their awareness to their thoughts and emotions without judgment. The process of being aware of the present moment without judgment can allow individuals to face negative thoughts or emotions without reacting to them automatically. By having that space where they are aware of their thoughts and emotions nonjudgmentally, they have a safe space where they change their usual way of reacting to such thoughts and emotions themselves. They can have the choice to be compassionate towards themselves in the process. By practicing self-compassion, they can then decrease negative self-judgment that can lead to painful emotional repercussions and increase self-support. In mindfulness practice, individuals are guided to decrease negative thought patterns, such as, “I cannot do this” or “I feel bad and feel the bad things.” [36], and instead say to themselves, “I cannot do this now, or I cannot do this yet, and that is alright”, “This is what I can do now”. By observing and accepting what is there in the moment reframes the perspective of the individual. By being aware of what is occurring internally, it can then provide a way for the individual to experience thoughts and feelings with warmth and kindness [37]. As researchers on mindfulness point out, being mindful prevents the automatic reaction to negative thoughts and emotions, thus allowing space to approach these with acceptance, curiosity, and compassion [20,37,38]. 

Participating in an MBCT program has been found to cultivate self-compassion [20,36]. In the second edition of the MBCT manual (as opposed to the first), it has been explicitly stated that MBCT aims to cultivate self-compassion [38]. In a study exploring MBSR and self-kindness, a component in self-compassion, an improvement was observed when utilizing regular 10-min mindfulness exercises [39,40]. Schroevers, Tovote, Snippe, and Fleer [41] found that adults who underwent MBCT have an increase in mindfulness and self-compassion after the program. In another study [36], part of their research involving MBCT was to analyze self-compassion, and it was discovered that after the MBCT program, adults with reported recurring depression had significant changes in self-compassion which in turn prevented relapses in depression. After participating in MBCT, other studies have also found significant changes in self-compassion among pregnant women [42], trainee clinical psychologists [43], as well as in students, adolescents, and adults in a community [20,44,45].

D. Empathy and Mindfulness

Empathy has been found to be another important competence in navigating college life. Rogers [46] defined empathy as ‘perceiving the internal frame of reference of another with accuracy, and with the emotional components and meanings which pertain thereto, as if one were the other person, but without ever losing the ‘as if’ condition’ (pp. 210–211). Through empathy, one learns to understand the subjective experiences and reality of the other person by sharing their perspective without getting lost in that person’s experiences [47]. Associations can be found between empathy and enhanced interpersonal connections and understanding. Empirically, the quality of interpersonal connections can be related to psychological and physical well-being in that being empathic also helps bring about improvement in interpersonal functioning [48,49]. Interaction with empathic curiosity and taking other people’s perspectives is a key part of adolescents’ development of identity and intimacy [50], which is both necessary in this stage, as well as a key factor for psychological well-being. Another importance of empathy with college students involves one of the most common issues in schools: bullying. Gagnon [51] has observed how there could be a relationship between empathy, aggression, and bullying. Lower empathy plays a role in the development and maintenance of bullying behavior. Lower empathy has also been associated with more aggression [52].

Among adolescents, empathy is necessary for interpersonal relationships [53,54,55]. Empathy helps adolescents understand others better by seeing another perspective and to have a deeper awareness of social situations. By being empathic, students can have healthier social interactions and better emotional competency with their peers [56]. In this generation where adolescents tend to be more detached and have less face-to-face interaction with others due to the high amount of time and energy spent on gadget use, empathy has been observed to drop among college students, most especially over the past decade [57]. Maintaining healthy interpersonal relationships with other people has been found to improve psychological health and well-being. Poor social connections and support from others have been associated with stress and affect mental health negatively [58]. Considering this negative development of empathy within adolescents, it is necessary to explore ways to help enhance empathy with adolescents.

There has been a significant amount of research on how the practice of mindfulness can promote an increase in empathy. Physiologically, it was discovered how practicing paying attention to one’s inner experience develops the prefrontal cortex, anterior cingulate cortex, and anterior insula, the same regions in the brain that are needed to improve empathy [59,60]. Another possible reason as to how mindfulness practice can affect empathy is through focused self-awareness. The dynamic of this was as follows: in becoming aware of one’s emotions, this leads to a better understanding of how they are occurring and what happens when they do. Being mindfully aware then helps them understand their own emotions as they arise, which then prevents them from getting caught up in these emotions. Once they are aware of the present moment experience and are not pulled away (by negativity, for example), individuals are able to be aware of another person’s experience, and what they must be feeling [45,61]. Similarly, another way mindfulness can affect empathy is through nonreacting and nonjudging, which are components of mindfulness. An individual can distance themselves from their own difficult emotions, and attend to the suffering other [62,63]. While there is plenty of literature to support the efficacy of mindfulness on empathy, there are studies that show inconsistencies about this as well. Ridderinkhof and colleagues [64], looked at the impact of a brief mindfulness practice on empathy in adults. Their results showed that mindfulness did not have a significant effect on empathy due to several possible factors, including the amount of mindfulness practice the participants had. 

MBCT, in particular, has been found to have an effect on empathy. Research has been discovering how being observant, rather than reactive, to one’s state of body and mind can build skills helpful for young people [65]. Studies that have explored how mindfulness-based interventions, in general, can affect empathy [61,66]. Schure, Christopher J., and Christopher S. [67] reported that after going through an adapted MBSR program, graduate students in psychology had increases in empathy. In a study by Block-Lerner, Adair, Plumb, Rhatigan, and Orsillo [61], participants who underwent a form of mindfulness-awareness exercise adapted from the work of Segal and colleagues, were discovered to have greater capacity to take the perspective of others in the group and to be more aware of the thoughts and feelings of others. Shapiro, Schwartz, and Bonner [66] found how medical students who participated in MBSR had improved empathy after the program. Several researches also explored how even brief mindfulness practices can improve empathy [64]. Though the results were not consistent with the body of literature, it offers the potential to continue exploring this direction. Through perspective taking (ability to take another person’s perspective) and empathic concern (feel concern for others), young people are more emotionally available to be present to the needs of their peers.

The increase in literature on mindfulness interventions suggests promising approaches that can be implemented in the Philippines. MBCT was developed as a low-cost intervention. As such, this is an intervention that Filipinos can benefit from. Given the developmental changes adolescents begin to undergo and the unfortunate increase in psychological difficulties, such as depression and suicide rates among Filipino students [68,69], implementing an adapted MBCT with Filipino students can be beneficial. Furthermore, research on how mindfulness can improve compassion to self and empathy to others will also be helpful. 

In this study, a mindfulness intervention (MBCT) was adapted and imbedded within a college counseling elective class. Prior to adapting MBCT in the classroom setting, the facilitator underwent rigorous training in MBCT. Within the classroom context, a pilot study was conducted to explore the effects of the mindfulness intervention on mindfulness, self-compassion, and empathy.

## 2. Materials and Methods

### 2.1. Design

This study used a quasi-experimental design with a control group. Convenience sampling was used to investigate the effect of an adapted mindfulness-based intervention embedded with a class of senior Psychology major college students. The participant sample was based on the voluntary enrollment of students that chose a counseling class with a mindfulness component and a counseling elective without a mindfulness component amidst other elective classes for that semester. Since the students enrolled in the particular class of their own choosing, they were all potential participants in the study. 

As part of ethical considerations, the study was guided by certain protocols of the university. Some of the ethics protocols are that survey forms students filled up did not include any identifying information. All records of their tests were kept in a secure location to ensure they were kept safe. The students were also informed of the voluntary nature of filling and completing the battery of tests. Testing was done by a graduate assistant without the professor present. All data were analyzed after the semester when grades were given out. The professor did not have any access to the survey data.

### 2.2. Participants

The participants of the study were Filipino Psychology Majors, in their senior year, ages 18–22 (M = 20.4, SD = 0.9). Students enlisted in one of two counseling-elective classes in psychology through an online enlistment process before the beginning of the semester. The first counseling class contained a mindfulness component, while the other counseling class did not have one based on the design of the class for that semester. Inquiry on whether the participants had engagement in any formal mindfulness practice at the beginning of the study was also established to check if any potential participant had a formal mindfulness practice before. There were 20 students enlisted in the mindfulness group, and 10 students in the control group. 

### 2.3. Measures

Five-Facet Mindfulness Questionnaire. The Five Facet Mindfulness Questionnaire (FFMQ), a 39-item questionnaire [70], was used to measure the students’ level of mindfulness before and after the intervention. The FFMQ is a measure of mindfulness commonly used to assess change before and after mindfulness-based interventions, such as MBCT and MBSR. The first subscale is Observing which refers to attending or noticing internal and external experiences (e.g., sounds, emotions, thoughts, bodily sensations, smells). Describing is a scale that measures the ability to express experiences of an individual in words. Acting with awareness measures the attending to one’s present moment activity, rather than being on “autopilot,” or behaving automatically, while attention is focused somewhere else. Nonjudging of inner experience is the scale that measures accepting without judging thoughts and emotions (e.g., as “good” or “bad”). Finally, Nonreactivity to Inner Experience refers to the ability to detach from thoughts and emotions, allowing them to come and go without getting involved or carried away by them. 

Each item is scored on a 5-point Likert scale ranging from 1 (never or very rarely true) to 5 (very often or always true). Baer, Smith, Hopkins, Krietemeyer and Toney, [70] suggest that it has reasonable psychometric properties, with a reliability of (0.92) and internal consistency for all facets in all samples were adequate-to-good (range 0.72 to 0.92) [71]. In this study, the reliability was found to be (0.69).

Self-Compassion Scale-Short Form. The Self-Compassion Scale (SCS) [72] is a 12-item questionnaire that measures self-compassion. The SCS was developed using an undergraduate sample and has been used constantly to measure self-compassion in literature. Strauss et al. [73] conclude that the SCS was the most valid and reliable measure of self-compassion compared to other measures. According to Neff and colleagues [74], the scale can be used to measure overall self-compassion levels, given that approximately 90% of item variance can be explained by a general self-compassion factor. 

Each item is scored on a 5-point Likert scale ranging from 1 (Almost Never) to 5 (Almost Always), which includes questions such as “When I’m going through a very hard time, I give myself the caring and tenderness I need”. The Self-Compassionate Scale [32] is the most widely used self-compassion measure in mindfulness-based interventions and MBCT research [35,73]. The SCS-Short Form proved to be reliable for use with a clinical college population, evidencing strong internal consistency (D = 0.85). The reliability observed in this study was almost identical to the reliability found for the original SCS-SF (D = 0.86) [72]. In this study, the reliability of this scale was found to be 0.90. 

Subscales in Interpersonal Reactivity Index. The Interpersonal Reactivity Index (IRI) [75] is a 28-item questionnaire that measures empathy through 4 subscales, perspective taking, fantasy, empathic concern, and personal distress. Empathy is defined as reactions of one individual to the observed experiences of another [75]. For this study, the scores from two scales were utilized. Empathic concern items assessed a tendency for the respondent to experience feelings of warmth, compassion, and concern for others undergoing negative experiences. In other words, this subscale measures other-oriented feelings of sympathy and concern for others in distress. Perspective-taking items reflected a tendency or ability of the respondent to adopt the perspective or point of view of other people. In other words, it assesses the tendency to spontaneously adopt the psychological point of view of others. Each item is scored on a 5-point Likert scale ranging from “Does not describe me well” to “Describes me very well”, which includes questions such as “Before criticizing somebody, I try to imagine how I would feel if I were in their place”. It has excellent psychometric properties and the instrument appears quite well-suited for use as a research tool in studying empathy and especially useful in investigations of the multi-dimensional nature of the empathic process [75]. Empathic Concern (EC) has a reliability of 0.88 and Perspective Taking (PT) has a reliability of 0.81. Validity for EC is 0.79, and PT is 0.77. In this study, reliability for Empathic Concern was 0.8, and for Perspective Taking, reliability was at 0.75.

### 2.4. Procedure 

In the latter part of the semester, before the mindfulness program began, the participants were given a battery of tests that measured self-compassion, empathy, and mindfulness a week before the intervention. Then, in the span of four weeks, in the counseling class with a mindfulness component, the students underwent the adapted form of the mindfulness-based cognitive therapy (MBCT) program [38], guided by a professor certified in MBCT and assisted by a graduate student. The mindfulness intervention was facilitated within class following the schedule in their semester, where classes took place twice a week. In the control group, classes continued based on their curriculum without a mindfulness component. All the students then answered the same group of tests a week after the completion of the mindfulness intervention.

### 2.5. Mindfulness Intervention

The mindfulness-based cognitive therapy curriculum was adapted through the duration of the sessions. The activities that were practiced can be found in Table 1. Notably, most of the program remained very similar to the original program. The program that was facilitated in the mindfulness counseling class was adapted in terms of the duration of each module. Furthermore, there were technical questions asked after each activity given that is still in a classroom setting with academic objectives to be fulfilled. Each session ran for 2 h, twice a week, in a span of 8 weeks. Furthermore, the program required the students to facilitate one practice each after they underwent all the activities as part of the class participation. These practices included body scan, sitting meditations, 3-min breathing space, mindful eating, and mindful stretching as part of teach-backs. Otherwise, the curriculum was consistent with the Segal, Williams, and Teasdale model.

## 3. Data Analysis

A comparison of means using a paired sample *t*-test research design was used to compare the effect of the mindfulness intervention on empathy, self-compassion, and mindfulness before and after the program. An advantage of using this form of statistical analysis is that fewer participants are needed in the data analysis. According to Howitt and Cramer [76], such a design only needs 20 participants, as opposed to independent groups, which would need 20 participants for each condition [76]. Using this design also decreases variability amongst the participants. Each participant undergoes the intervention. Therefore, it is expected that any changes from condition to condition are due to the nature of the intervention and not variability among participants. 

To compute for the effect size for each measure, Cohen’s D was used. Effect sizes assess the magnitude or the strength of the findings that occur in a research study. According to Cohen [77], a score of d = 0.2 would be considered a ‘small’ effect size, 0.5 represents a ‘medium’ effect size, and 0.8 suggests ‘large’ effect size.

## 4. Results

### 4.1. Mindfulness

There was a significant difference in the scores on the FFMQ (Table 2) before the mindfulness program (M = 2.86, SD = 0.52) and after the mindfulness program (M = 3.37, SD = 0.39); t (19) = −4.74, *p* < 0.05 with an increase of 0.51 (SD = 0.49) and a large effect size (d = 1.04). The subscales of FFMQ also had significant differences before and after the program. The Nonreactivity to inner experiences subscale had a significant difference before (M = 2.62, SD = 0.61) and after (M = 3.21, SD = 0.51); t (19) = −6.25, *p* < 0.05 with an increase of 0.59 (SD = 0.42) and a large effect size (d = 1.39). 

The Observing subscale had a significant difference before (M = 3.03, SD = 0.63); and after (M = 3.57, SD = 0.49); t (19) = −3.29, *p* < 0.05 with an increase of 0.54 (SD = 0.74) and a moderate effect size (d = 0.73). The Describing subscale had a significant difference before (M = 3.20, SD = 0.89) and after (M = 3.50, SD = 0.81); t (19) = −2.59, *p* < 0.05 with an increase of 0.31 (SD = 0.53) and a moderate effect size (d = 0.58). 

The Acting with awareness subscale had a significant difference before (M = 2.84, SD = 0.88) and after (M = 3.26, SD = 0.57); t (19) = −2.76, *p* < 0.05 with an increase of 0.41 (SD = 0.67) and a moderate effect size (d = 0.61). The Nonjudging to inner experiences subscale had a significant difference before (M = 2.55, SD = 0.94) and after (M = 3.19, SD = 0.98); t (19) = −2.42, *p* < 0.05 with an increase of 0.64 (SD = 1.19) and a moderate effect size (d = 0.54). 

There was no significant difference in the scores on the FFMQ (Table 3) in the pretest (M = 3.07, SD = 0.47) and in the posttest (M = 3.25, SD = 0.3); t (9) = −1.84, *p* > 0.05 for the control group.

### 4.2. Self-compassion

There was a significant difference in the scores for Self-Compassion (Table 2) before the mindfulness program (M = 2.62, SD = 0.82) and after the mindfulness program (M = 3.70, SD = 0.62); t (19) = −5.36, *p* < 0.05 with an increase of 1.09 (SD = 0.91) and a large effect size (d = 1.19). 

There was also a significant difference in the scores for Self-Compassion (Table 3) at pretest (M = 3.18, SD = 0.51) and posttest (M = 3.28, SD = 0.53); t (9) = −2.33, *p* < 0.05 with an increase of 0.10 (SD=) and a small effect size (d = 0.19) in the control group.

### 4.3. Empathy

Empathy was measured using the Empathic Concern and Perspective Taking subscales of the Interpersonal Reactivity Index (Table 2). There was a significant difference in the scores for Empathic Concern before the mindfulness program (M = 2.81, SD = 0.90) and after the mindfulness program (M = 3.22, SD = 0.55); t (19) = −2.55, *p* < 0.05 with an increase of 0.41 (SD = 0.73) and it had a moderate effect size (d = 0.56). 

There was a significant difference in the scores for Perspective Taking before the mindfulness program (M = 3.06, SD = 0.56) and after the mindfulness program (M = 3.34, SD = 0.24); t (19) = −2.10 *p* < 0.05 with an increase of 0.29 (SD = 0.61) and it had a small effect size (d = 0.48). 

There was no significant difference in the scores (Table 3) for Empathic Concern during pretest (M = 3.20.SD = 0.49) and in the posttest (M = 3.17, SD = 0.49); t (9) = 0.45, *p* > 0.05 for the control group. There was also no significant difference in the scores for Perspective Takin in the pretest (M = 2.86, SD = 0.79) and in the posttest (M = 2.87, SD = 0.63); t (9) = −0.10, *p* > 0.05 for the control group.

These results suggest that a mindfulness intervention does change one’s level of mindfulness and has a significant effect on empathy and self-compassion. These findings have implications for introducing mindfulness programs to a college student population.

## 5. Discussion

The results of this exploratory study suggest that practicing mindfulness can help college students. This study corroborates with Western studies about the efficacy of mindfulness practice for a young population, and how mindfulness can improve empathy and self-compassion. 

In the adapted mindfulness program, students were guided to have awareness of the present moment without judgment. Through this kind of awareness, they can avoid being overwhelmed and getting caught in intense situations [64]. This kind of awareness also leads to an increased nonjudgmental awareness of oneself and others. Guiding students to gently observe their thoughts and emotions as they occur in the present moment can influence self-compassion and empathy. 

Guiding the students to be aware of their experiences without judgment allowed them the space to be kind to themselves. As early as the practice of the breathing space in session 3, it provided the opportunity for students to practice take a pause and observe their inner experience in the moment. Students were led to connect to the present moment by stepping out of automatic pilot to ask, “Where am I? What’s going on?” Afterward, they brought their attention to a single object—their breath—which offers a change in the quality of awareness that can allow them to have a wider view of their experiences. As Neff described self-compassion as an awareness with a sense of warmth, compassion, and kindness, the awareness brought by the Breathing Space exercise might help to facilitate a softening of the experience of difficulty and to bring reassurance to them that what every they experience is okay. Furthermore, later exercises, such as longer sitting meditations and the Sitting with Difficulty exercise, might have helped develop a relationship with unwanted feelings by allowing difficult thoughts without judgment. By facing them without reacting and judgement, it was possible that a conscious choice was created to let go of unhealthy ways of thinking. In the exercise, Thoughts are not Facts, it allowed the students to address intense emotions that can affect thoughts and beliefs. It offers students the tools to distance themselves from negative thoughts by viewing them as mere mental events that they can view with gentle awareness and curiosity. By having a healthier way of responding to such thoughts, unhealthy reactions, such as harsh self-criticism, can be prevented, and it prevents them from being overwhelmed. Furthermore, session 7 of the program allowed the students to develop strategies of how to take care of themselves. They became aware of the patterns in their lives, particularly of activities that nourish and deplete them. In this session, they learned to practice self-care, how to respond differently in a more balanced way, and increase behaviors that can help bring more energy. In doing so, they became more conscious about treating themselves with warmth, kindness, and acceptance. 

In the same way, the program had particular exercises that could have influenced empathy. As early as Session 2 of the program, in the exercise, “Walking down the street”, the facilitators invited the students to become aware and to decenter from their usual patterns of thinking and feeling. This exercise presents a situation where the students pass by someone they know and do not acknowledge them. This outcome provided different responses from the students on what they thought and felt. In this exercise, an awareness of their thoughts involving other people was observed. This exercise opened the door to a wider perspective as they became aware of their thoughts and how those thoughts can sometimes be judgmental, and which can then lead to negative feelings of anger, worry, or concern. This exercise can facilitate understanding of others better by looking at experiences from a distance, with spacious awareness, and with interest and curiosity. This particular skill of mindfulness was further practiced in sessions 3 and 4 which included activities such as mindful seeing, mindful walking, and guided sitting meditations. Through these exercises, the students began to learn how to anchor their attention on a chosen point of focus. Doing this potentially decreased the possibility of becoming distracted or overwhelmed by their inner thoughts and emotions. Mindful seeing, in particular, invited the students to use their senses to be aware of their surroundings without judging them and just observing them just as they are. These mindfulness exercises guided students to be aware of their inner world and, at the same time, learn to expand their awareness without judgment or reacting to them. As studies show, the practice of mindfulness helps individuals to expand their awareness of another person’s experience, and what they must be feeling [40,61,78]. 

One of the unique activities in the adapted program was a particular class requirement called teach-backs. After undergoing a mindfulness exercise, they were given the opportunity to study it, understand its intention, and how it was facilitated. Afterward, they facilitated it in the class and were feedback on their facilitation. This could also have created a context where the students were more driven to understand and apply what they have learned.

Furthermore, as senior students, they were in their last year in college and, as such, might be said to have become more mature in terms of academic goals, personality traits, and psychological competencies [79,80]. As they get closer to graduation, they can become more engaged academically. Their cognitive capacities can be different from that of freshmen or sophomores. Senior students can have more developed abstract processing skills, improved ways of relating to others, and internal motivation.

In this study, the facilitator was also the professor of the class. With the dual roles of a facilitator and a professor, the nature of being a professor can influence the facilitation of the program. There are goals to be accomplished and lessons to be taught in class, as part of a classroom setting context. During the duration of the program, the facilitator might have guided the students in a more didactic way, which could have allowed a unique kind of learning and understanding of mindfulness concepts, different from a facilitator of a traditional MBCT intervention. Interest in exploring mindfulness interventions in a classroom setting is starting to grow and begin to acknowledge the promising potential [81]. One study explored how a learning system integrated with a mindfulness component can facilitate better outcomes that yielded significant results [82]. Another study by Schonert-Reichl et al. [83] investigated mindfulness in the classroom and found positive outcomes that prompt further research on the potential of mindfulness interventions with children and adolescents. 

In terms of future directions, more rigorous research should be conducted, particularly using a random sampling method and with more long-term studies with longer data collection points. Qualitative data can also be collected from participants to observe and describe their particular phenomenological experiences and better understand the underlying influences of mindfulness on self-compassion and empathy. Furthermore, studies on how mindfulness interventions can influence other capacities necessary to survive and thrive college life can also be recommended. Furthermore, it would be interesting to analyze specific factors in the tests of mindfulness, empathy, and self-compassion with different students and other members of the academe. There are subscales found in the IRI scales, SCS, and FFMQ that can be further explored as part of mindfulness research. The efficacy of mindfulness can also be studied across different groups of students, including more high-risk populations (students diagnosed with psychological disorders), special populations (students with Attention deficit hyperactivity disorder or Autism spectrum disorder), or other particular samples (athletes or leaders).

## 6. Limitations of the Study

One major limitation of this study was that the participants of the study were enrolled in a specific class, and thus, participation was a nonrandomized sampling. While the statistics suggest the efficacy of the adapted program, results should also take other factors into consideration. First, the program was embedded in a counseling class for seniors. This meant that were lectures that could have explained theories related to the program that influenced their thought processes and, consequently their level of engagement. Being a class in college, there were requirements, such as final examinations and papers to be submitted, that could also have increased participation and deepened their motivation to learn. Another factor that could have influenced the results was the nature participants themselves. In this study, the participants were Psychology majors in their senior year. Due to the nature of their course, there could have been a familiarity with psychology concepts and processes as they have encountered them in their different lectures and classes. Another limitation was that the sample size for a class was not large. Due to the study being embedded in a class, there could have been different factors that may have influenced the results of the study. The teacher of the class, who was also the facilitator of the program, might have different effects on the students. Since the facilitator of the program was also the professor of the students in the class, this could have lead the students to engage themselves more with aims to do well in the class. Students’ participation could have also been different if the program was implemented outside of the classroom setting. Since the program was embedded in a class, the students could have been influenced to participate more considering their grades for that class. There was no control group present that could provide comparisons for mindfulness, self-compassion, and empathy. These factors could have affected the outcomes of the study. 

This study explored how incorporating mindfulness in the education of college students in the Philippines may be helpful. An adapted MBCT program was administered to Filipino Psychology Majors, and after going through the program, it was observed that they had an increase in mindfulness, empathy, and self-compassion. Overall, this study suggests how mindfulness practice may be integrated to help college students cope with their stresses and challenges, by being kinder to themselves and empathic to others, through becoming more mindful of their inner experience with acceptance and non-judgment. 

## Figures and Tables

**Table 1:** Adapted mindfulness-based cognitive therapy.

Timetable of Sessions	Activities of Session
Session 1: Awareness and Automatic Pilot	Raising Exercise and Body Scan Homework: Body Scan and Mindfulness Activities
Session 2: Living in Our Heads	Body Scan, 10 Minute Sitting Meditation, Thoughts, and Feelings Exercise “Walking Down the Street” Homework: Body Scan, Pleasant Events Calendar, and Mindfulness Activities
Session 3: Gathering the Scattered Mind	Mindful Seeing Exercise, 30 Minute Sitting Meditation, Mindful Movement “Mindful Stretching” Homework: Mindful Movement, 3-min Breathing Space, Unpleasant Events Calendar, and Mindfulness Activities
Session 4: Recognizing Aversion	Mindful Walking, 40 Minute Sitting Meditation, 3 Minute Breathing Space Homework: Sitting Meditation, Mindful Walking, 3-min Breathing Space, and Mindfulness Activities
Session 5: Allowing	Working with the Difficulty Meditation, 3-Minute Breathing Space Homework: Working with Difficulty meditation, and 3-min Breathing Space
Session 6: Thoughts are not Facts	40 Minute Sitting Meditation, Breathing Space, Relapse Prevention Activity Homework: Any formal Mindfulness practice they choose, and 3-min Breathing Space
Session 7: Taking care of Self	30 Minute Sitting Meditation, Taking Care of Self Exercise, Activity and Mood Exercise, Nourishing and Depleting Exercise, Mindful Walking Homework: Body Scan, 3-min Breathing Space and Any Formal Mindfulness practice they choose
Session 8: Maintaining and Extending	Body Scan

**Table 2:** Means, *p*-value, and effect size of the mindfulness class.

		Mean	N	Mean Difference	*p* Value	Effect Size
FFMQ (mindfulness)	Before	2.86	20	0.51	*p* < 0.05	d = 1.04
	After	3.37				
SCS (self-compassion)	Before	2.62	20	1.09	*p* < 0.05	d = 1.19
	After	3.70				
EC (empathic concern)	Before	2.81	20	0.41	*p* < 0.05	d = 0.56
	After	3.22				
PT (perspective taking)	Before	3.06	20	0.29	*p* < 0.05	d = 0.48
	After	3.34				

**Table 3:** Means, *p*-value, and effect size of the control group.

		Mean	N	Mean Difference	*p* Value	Effect Size
FFMQ (mindfulness)	Before	3.07	10		*p* > 0.05	
	After	3.25				
SCS (self-compassion)	Before	3.18	10		*p* < 0.05	d = 0.19
	After	3.28		0.10		
EC (empathic concern)	Before	3.20	10		*p* > 0.05	
	After	3.17				
PT (perspective taking)	Before	2.86	10		*p* > 0.05	
	After	2.87				
